# Manure-biochar compost mitigates the soil salinity stress in tomato plants by modulating the osmoregulatory mechanism, photosynthetic pigments, and ionic homeostasis

**DOI:** 10.1038/s41598-024-73093-5

**Published:** 2024-09-20

**Authors:** Mohammed Zia Uddin Kamal, Umakanta Sarker, Siddhartha Kumar Roy, Mohammad Saiful Alam, Mohammad Golam Azam, Md. Yunus Miah, Nazmul Hossain, Sezai Ercisli, Saud Alamri

**Affiliations:** 1 Department of Soil Science, Faculty of Agriculture, Bangabandhu Sheikh Mujibur Rahman Agricultural University (BSMRAU), Gazipur-1706, Bangladesh; 2 Institute of Climate Change and Environment, BSMRAU, Gazipur, 1706 Bangladesh; 3 Department of Genetics and Plant Breeding, Faculty of Agriculture, Bangabandhu Sheikh Mujibur Rahman Agricultural University (BSMRAU), Gazipur-1706, Bangladesh; 4 Khulna Public College, Boyra, Khulna Bangladesh; 5 Pulses Research Centre, Bangladesh Agricultural Research Institute, Ishurdi, 6620 Bangladesh; 6 Department of Agronomy, Iowa State University, Iowa, Ames, 50010 USA; 7 Department of Horticulture, Faculty of Agriculture, Ataturk University, Erzurum, 25240 Türkiye; 8 Department of Botany and Microbiology, College of Science, King Saud University, Riyadh, Saudi Arabia

**Keywords:** Growth, Yield, Organic amendments, Salinity, Tomato, Salt, Abiotic

## Abstract

One of the main abiotic stresses that affect plant development and lower agricultural productivity globally is salt in the soil. Organic amendments, such as compost and biochar can mitigate the opposing effects of soil salinity (SS) stress. The purpose of this experiment was to look at how tomato growth and yield on salty soil were affected by mineral fertilization and manure-biochar compost (MBC). Furthermore, the study looked at how biochar (organic amendments) work to help tomato plants that are stressed by salt and also a mechanism by which biochar addresses the salt stress on tomato plants. Tomato yield and vegetative growth were negatively impacted by untreated saline soil, indicating that tomatoes are salt-sensitive. MBC with mineral fertilization increased vegetative growth, biomass yield, fruit yield, chlorophyll, and nutrient contents, Na/K ratio of salt-stressed tomato plants signifies the ameliorating effects on tomato plant growth and yield, under salt stress. Furthermore, the application of MBC with mineral fertilizer decreased H_2_O_2_, but increased leaf relative water content (RWC), leaf proline, total soluble sugar, and ascorbic acid content and improved leaf membrane damage, in comparison with untreated plants, in response to salt stress. Among the composting substances, T_7_ [poultry manure-biochar composting (PBC) (1:2) @ 3 t/ha + soil-based test fertilizer (SBTF)] dose exhibited better-improving effects on salt stress and had maintained an order of T_7_ > T_9_ > T_8_ > T_6_ in total biomass and fruit yield of tomato. These results suggested that MBC might mitigate the antagonistic effects of salt stress on plant growth and yield of tomatoes by improving osmotic adjustment, antioxidant capacity, nutrient accumulation, protecting photosynthetic pigments, and reducing ROS production and leaf damage in tomato plant leaves.

**Supplementary Information:**

The online version contains supplementary material available at 10.1038/s41598-024-73093-5.

## Introduction

One of the worst environmental pressures that seriously jeopardize agricultural sustainability and productivity is salinity^1.2^. The salinity of the soil is a worldwide issue that affects both developed and developing nations^3^. Future climate change scenarios are anticipated to worsen the salinity-induced deterioration of soil. These scenarios include increasing sea levels and their impacts on coastal regions, increasing temperatures, and rising evaporation. Soil salinization^4^ affects approximately 1,128 million hectares of land worldwide, and secondary salinization has added 77 million hectares of land^5^. Approximately 20% of all farmed and 33% of irrigated agricultural fields globally are affected by soil salinization^6^. Estimates of the global distribution of regions impacted by salt indicate that 50.8 million hectares are in Europe and 87.6 million hectares are in South Asia^7^. Comparably, 30% of Bangladesh’s farmland area roughly one million hectares is damaged by salinization to varied degrees^8,9^. Salinity-induced losses in crop production cause irrigated agriculture to lose USD 27.2 billion annually in economic value. Abiotic stress, such as salinity/drought simultaneously causes ion toxicity, osmotic stress^10^, and oxidative stress^11,12^, which affect the physiological and biochemical processes of many plants, including photosynthesis^13^, energy metabolism, plant water, nutrient uptake imbalance^14^, protein^15^, DNA, membrane damage^16^, etc., and ultimately affect the growth^17^ and yield of crops^18^. Osmotic stress in plants changes many enzymatic antioxidants, such as CAT, SOD, GOPX^11^, non-enzymatic antioxidants, such as carotenoids^19,20^, betalains^21,22^, ascorbic acids^23,24^, betaxanthins^25,26^, chlorophyll *a*^27^, betacyanins^28^, chlorophyll *b*^29^, xanthophylls^30^, beta-carotene^31^, phenolic and flavonoids, such as hydroxycinnamic acids^32^, hydroxybenzoic acids^33^, flavanols^34^, flavones^35^, flavanones^36^, flavonols^37^, etc. having high antiradical capacities^38^ which can manage the adverse impact of abiotic stresses. Under abiotic stress, the plant itself regulates different pathways to increase the application of these antioxidants^39^ to detoxify the ROS^40^ and mitigate environmental stress.

Furthermore, SS poses a serious risk to farmland soil production by adversely affecting microbial activity, nutrient availability, and soil characteristics^41,42^. There is usually a 30–50% yield loss, depending on the SS^43^. Plants have developed a variety of defense mechanisms to withstand salinity, including ion homeostasis and compartmentalization, osmoprotectant production, antioxidant activation, and the control of several stress-sensitive genes^44^. Various reclamation techniques, including chemical remediation, phytoremediation, soil water leaching, organic amendment, etc., can be used to improve the congenial environment that plants use to withstand stress. Thus, reducing the salt burden on crop growth in appropriate and economical ways has become a top concern in the development of technologies to guarantee global food security.

In salinity-degraded soil, soil management and agricultural production techniques depend on maintaining a suitable level of soil organic matter and guaranteeing the effective biological cycling of nutrients^45^. Organic amendments may be considered a sustainable method of lessening the effects of salinity stress on crops since they have both ameliorative effects and enhance the fertility status of saline soils^46^. Salinity stress can have significant effects on various crops. A high soil salinity level can lead to water stress and ion toxicity, decreasing water availability and increasing the rate of transpiration in plants^47^. In addition, high soil salinity causes ion homeostasis to be disrupted, resulting in an accumulation of toxic ions such as sodium and chloride in plant tissues^48^. According to several studies, organic matter’s improved physical, chemical, and biological qualities could help reduce salt stress^49,50^. However, adding organic matter won’t be able to sustainably improve this deteriorated soil in the tropics because of the natural quick degradation of organic matter and poorly stable chemicals^51^. Almond shell-derived biochar (ASB) induced changes in soil microbial composition and communities^52^. The use of more stable carbon compounds, such as carbonized materials, is an alternative to organic additions. Because of its strong Na^+^ adsorption potential and relatively stable nature, the biomass product known as “biochar,” which is produced through anaerobic or limited oxygen condition pyrolysis, may be able to reduce salinity stress^53,54^. Because of its increased ability to add Ca^2+^ and Mg^2+^, improve the physical characteristics of the soil, including aggregate stability, porosity, saturated hydraulic conductivity, and bulk density, and consequently improve Na^+^ leakage, biochar becomes an efficient remediation approach in salty soil^55^. However, due to its expensive and unusual production methods, there is limited data available on the practice of biochar as a viable soil additive for salty soils. For agriculture that is susceptible to salinity stress, appropriate and economical methods of minimizing the negative effects of salt stress on crop growth have thus become crucial. The study reported that biochar-manure compost (poultry manure mixed with biochar (1:3) in conjunction with pyroligneous solution had significant positive effects on salinity reduction in cereals like maize productivity^54^. However, further research is needed to elucidate the effects of different manure and biochar compost combinations on salt-sensitive vegetables, such as tomatoes. Thus, we hypothesize that composting manure and biochar can reduce salt stress and will be a cost-effective strategy for tomato growers in saline-sensitive locations. Different crops have varying levels of salt tolerance. Some crops, like barley, sugar beet, and spinach, are more salt-tolerant and can withstand higher levels of salinity. On the other hand, crops such as tomatoes, eggplant, beans, and many fruit trees are more sensitive to salt stress^56^.

The majority of Bangladesh’s salinity-affected areas experience seasonal flooding from salt water, which varies the degree of salinization of the soil. The majority of the land remains fallow during the dry season because of the elevated salinity of the soil and the shortage of high-quality water^57^. One of the most significant and nutrient-dense foods grown in arid, salinity-degraded agricultural regions maybe tomatoes. The Solanaceae family includes the tomato (*Lycopersicon esculentum* L.), most of which are cultivated in nearly every part of the globe. It ranks as the second most significant crop among the 24 vegetable crops. Tropical, subtropical, and even temperate regions of the world are where it is primarily farmed^58^. Due to their flavor and nutritional worth, tomatoes are an essential part of the human diet. Because of its short root structure, high stomatal conductance, and wide diversity of leaf types, the tomato plant is the most vulnerable to salt stress^59^. If we are to convert a large number of saline soils under circumstances of intensive agriculture and adapt to climate change, we must comprehend the impacts of salt stress and find measures to decrease it. There is scarce information on the effects of salt stress on tomatoes as well as the affordable and practical methods of lowering the effects of salt by organic amendment. This study aims to assess the possibility of mitigating SS stress with the addition of compost made of manure and biochar, as well as the mechanisms involved.

## Results

The effects of different fertilizers - biochar compost and organic materials on tomato plants under SS stress were studied, including vegetative development, photosynthetic pigmentation, oxidative damage, and their osmotic adjustment properties, leaf nutrient content, biomass, and fruit yield characteristics.

### Growth traits

**Fig. 1:**
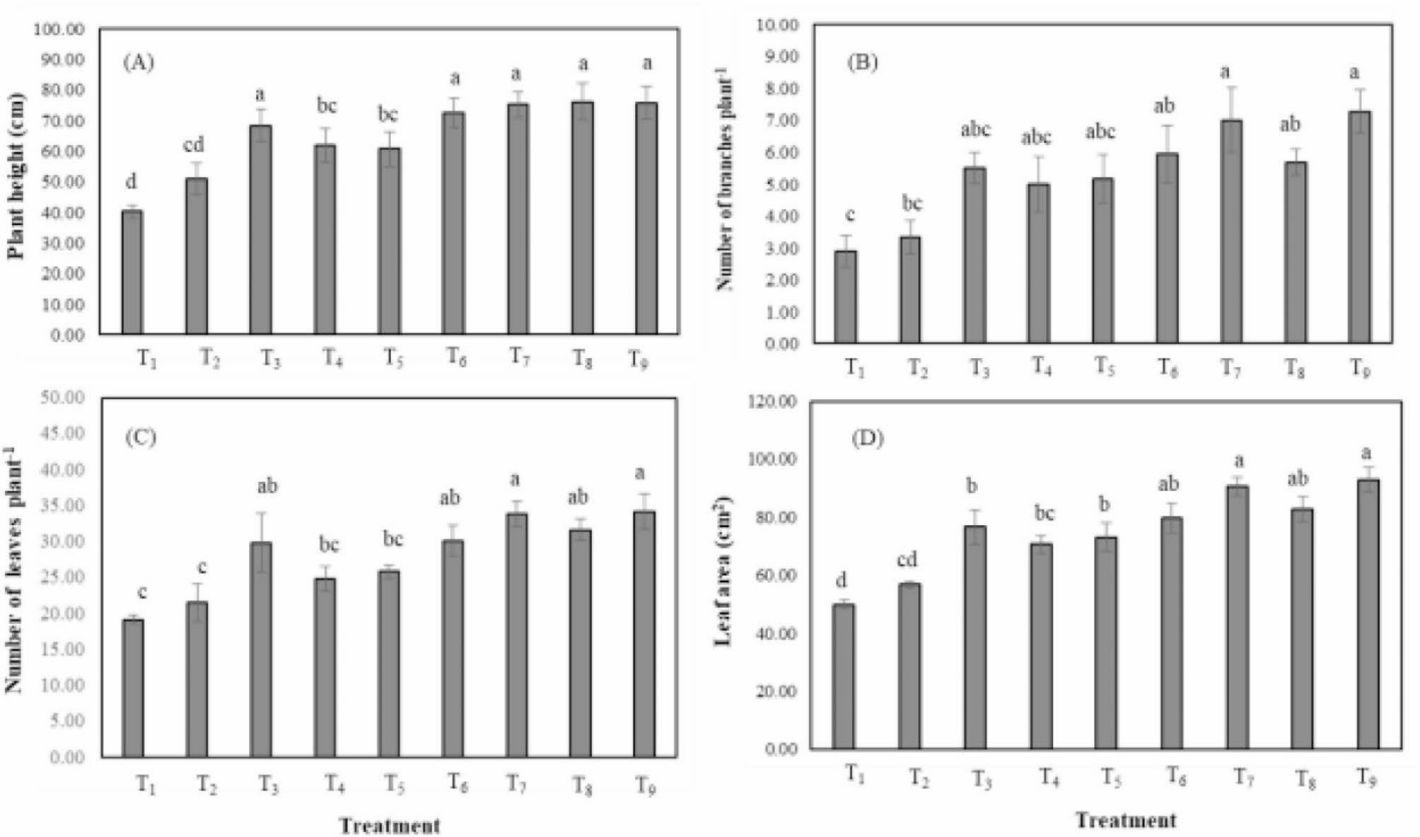
Manure and biochar compost (MBC) application impacts on salt-stressed tomato plant growth **(A)** PH, **(B)** NBP, **(C)** NLP, and **(D)** LA (cm^2^). The value indicates the mean (± SD); *p* < 0.05; *n* = 9; Different letters indicate significant differences among treatments (*p* < 0.05) by Tukey’s HSD test. Treatments combinations are T_1_ = SS; T_2_ = soil-based test fertilizer (SBTF); T_3_ = B @ 3 t/ha + SBTF; T_4_ = P @ 3 t/ha + SBTF; T_5_ = C @ 3 t/ha + SBTF; T_6_ = poultry manure-biochar composting (PBC) (1:1) @ 3 t/ha + SBTF; T_7_ = PBC (1:2) @ 3 t/ha + SBTF; T_8_ = cow dung-biochar composting (CBC) (1:1) @ 3 t/ha + SBTF; T_9_ = CBC (1:2) @ 3 t/ha + SBTF.

Tomato plants grown in untreated saline soil had lower plant height (PH) (40.20 cm), number of branches per plant (NBP) (2.89), number of leaves per plant (NLP) (19.17), and leaf area (LA) (49.90 cm^2^), hence exhibiting significant inhibition of plant growth (Fig. 1). The application of different manures, rice husk biochar, and their composting significantly improved the growth characteristics of salt-stressed tomato plants (Fig. 1).  

Treatment T_8_ [cow dung-biochar composting (CBC) (1:1) @ 3 t/ha + SBTF] showed the highest PH (76.09 cm) and statistically similar data in T_3_ (B @ 3 t/ha + SBTF), T_6_ (poultry manure-biochar composting (PBC) (1:1) @ 3 t/ha + SBTF), T_7_ [PBC (1:2) @ 3 t/ha + SBTF] and T_9_ [CBC (1:2)) @ 3 t/ha + SBTF] treatment (Fig. 1A). Treatments of T_2_, T_3_, T_4_, T_5_, T_6_, T_7,_ T_8_, and T_9_ increased PH by 26.83%, 69.92%, 50.50%, 51.00%, 80.27%, 84.34%, 91.26%, and 86.58%, respectively, compared with T_1_ treatment (Table 1). Manure-biochar compost treatment T_9_ showed the maximum NBP (7.27), and responses were similar for all organic amendments (Fig. 1B). No significant changes in NBP were found after a single fertilization compared to the control NBP increased by 96.30-151.73% in MBC treatments compared to the control (Table 1). Meanwhile, the T_9_ treatment showed maximum leaf proliferation i.e. NLP (34.17) and LA (92.77 cm^2^) (Fig. 1C). Organic amended plants showed more NLP i.e., in T_3_ (55.65%), T_4_ (26.05%), T_5_ (34.87%), T_6_ (57.39%), T_7_ (76.61%), T_8_ (65.22%) and T_9_ (78.26%) than plots with untreated saline soil (Table 1). Likewise, the result in Fig. 1D revealed that LA of organic amended salt-stressed plants increased in T_3_ (53.49%), T_4_ (39.68%), T_5_ (46.45%), T_6_ (59.73%), T_7_ (81.77%), T_8_ (65.78%) and T_9_ (85.92%) compare to T_1_ (Table 1).

**Table 1:** Percent change in growth, yield, and yield attributes of tomato plants treated with manure and biochar compost in salt-stressed soil compared to untreated control.

Parameters	Treatments							
	T_2_	T_3_	T_4_	T_5_	T_6_	T_7_	T_8_	T_9_
PH	26.83	69.92	50.50	51.00	80.27	84.34	91.26	86.58
NBP	15.47	90.53	55.88	78.98	105.54	142.49	96.30	151.73
NLP	12.17	55.65	26.05	34.87	57.39	76.61	65.22	78.26
LA	13.83	53.49	39.68	46.45	59.73	81.77	65.78	85.92
NEFC	17.95	64.10	15.38	43.59	94.87	102.05	130.77	128.21
NFC	6.96	23.41	29.11	18.99	40.38	42.91	56.96	48.73
NFP	24.83	100.00	47.83	68.50	170.02	186.65	260.97	238.00
SFW	15.50	24.91	29.24	19.69	23.87	27.58	31.21	28.54
FWP	44.50	150.49	91.74	103.83	235.40	264.62	374.53	337.69
FY	44.50	150.49	91.74	103.83	235.40	264.62	374.53	337.69
TDM	38.34	88.47	56.98	74.94	92.67	121.69	107.22	117.98

PH = Plant height, NBP = Number of branches per plant, NLP = Number of leaves per plant, LA = Leaf area, NEFC = Effective flower cluster per plant, NFC = Number of fruit per cluster, NFP = Number of fruit per plant, SFW = Single fruit weight, FWP = Fruit weight per plant. FY = Fruit yield, TDM = Total dry mass. Treatments combinations are T 1  = SS; T 2  = SBTF; T 3  = B @ 3 t/ha + SBTF; T 4  = P @ 3 t/ha + SBTF; T 5  = C @ 3 t/ha + SBTF; T 6  = PBC (1:1) @ 3 t/ha + SBTF; T 7  = PBC (1:2) @ 3 t/ha + SBTF; T 8  = CBC (1:1) @ 3 t/ha + SBTF; T 9  = CBC (1:2) @ 3 t/ha + SBTF.

### Yield and yield features

**Fig. 2:**
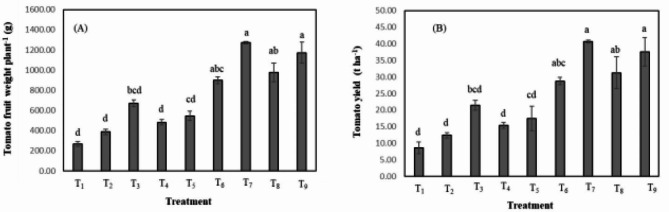
Effects of manure and biochar compost (MBC) on (**A**) tomato fruit weight plant^−1^ (g) and (**B**) tomato yield (t/ha) of tomato plant grown in saline soil. The value indicates the mean (± SD); *p* < 0.05; *n* = 9; Different letters indicate significant differences among treatments (*p* < 0.05) by Tukey’s HSD test. Treatments combinations are T_1_ = SS; T_2_ = SBTF; T_3_ = B @ 3 t/ha + SBTF; T_4 =_ P @ 3 t/ha + SBTF; T_5_ = C @ 3 t/ha + SBTF; T_6_ = PBC (1:1) @ 3 t/ha + SBTF; T_7_ = PBC (1:2) @ 3 t/ha + SBTF; T_8_ = CBC (1:1) @ 3 t/ha + SBTF; T_9_ = CBC (1:2) @ 3 t/ha + SBTF.

Tomato fruit yield and yield traits were calculated on abandoned salt-stressed (control) plants and various organic amended plants (Table 2; Fig. 2). Yield-promoting parameters of salt-stressed tomato plants were significantly influenced by the application of manure-biochar compost, its raw material plus soil test-based (SBTF) fertilizer (Table 2). Plants grown under salt stress (control treatment) showed the lowest number of effective flower cluster plant^−1^ (NEFC) (3.25), number of fruit cluster^−1^ (NFC) (2.57), number of fruit plant^−1^ (NFP) (8.63), and single fruit weight (SFW) (31.18 g). In the T_9_ treatment, the maximum NEFC (7.50), NFC (4.13), NFP (31.13), and SFW (40.92 g) were recorded. Notably, NEFC, NFC, NFP, and SFW increased significantly by 94.87–130.77%, 40.38–56.96%, 170.02–260.97%, 23.87–31.21%, respectively under the MBC treatments over the absolute saline soil plants (Table 1). The manure-biochar compost treatments showed better yield traits data than the cow dung, poultry manure, and biochar alone.

**Table 2:** Manure and biochar compost (MBC) application impacts on yield and yield attributes of salt-stressed tomato plant.

Treatments	NEFC	NFC	NFP	SFW (g)
T_1_	3.25 ± 0.25 e	2.63 ± 0.40 d	8.61 ± 1.07 c	31.18 ± 1.05 b
T_2_	3.83 ± 0.28 de	2.82 ± 0.16 cd	10.77 ± 0.25 c	36.02 ± 3.28 ab
T_3_	5.33 ± 0.58 bcd	3.25 ± 0.25 abcd	17.25 ± 0.90 bc	38.95 ± 2.57 ab
T_4_	4.48 ± 0.64 de	3.05 ± 0.32 bcd	13.55 ± 0.83 c	35.82 ± 4.25 ab
T_5_	4.67 ± 0.58 cde	3.13 ± 0.31 bcd	14.53 ± 1.26 c	37.32 ± 4.80 ab
T_6_	6.33 ± 0.50 abc	3.70 ± 0.35 abc	23.29 ± 0.89 ab	38.63 ± 2.00 ab
T_7_	7.50 ± 0.68 a	4.13 ± 0.35 a	31.13 ± 5.23 a	40.92 ± 1.53 a
T_8_	6.57 ± 0. 48ab	3.76 ± 0.06 abc	24.72 ± 2.38 ab	39.78 ± 4.12 ab
T_9_	7.42 ± 0. 52a	3.92 ± 0.39 ab	29.15 ± 4.60 a	40.08 ± 1.45 ab
Significance level	< 0.001^***^	< 0.001^***^	< 0.001^***^	< 0.043^*^

The value indicates the mean (± SD); *p* < 0. 05; n  = 9. Different letters indicate significant differences among treatments ( p  < 0.05) by Tukey’s HSD test. Treatments combinations are T 1  = SS; T 2  = SBTF; T 3  = B @ 3 t/ha + SBTF; T 4  = P @ 3 t/ha + SBTF; T 5  = C @ 3 t/ha + SBTF; T 6  = PBC (1:1) @ 3 t/ha + SBTF; T 7  = PBC (1:2) @ 3 t/ha + SBTF; T 8  = CBC (1:1) @ 3 t/ha + SBTF; T 9  = CBC (1:2) @ 3 t/ha + SBTF; . NEFC = Effective flower cluster per plant, NFC = Number of fruit per cluster, NFP = Number of fruit per plant, SFW = Single fruit weight.

No significant changes in tomato plant yield characteristics were observed with absolutely SBTF-based fertilization compared to unamended saline soils.

The data in Fig. 2 showed that the treatments of different organic amendments have a significant positive effect on the fruit weight plant^−1^ (FWP) and the total fruit yield of salt-stressed tomato plants. The T_7_ treatment of poultry manure-biochar compost (1:2) recorded the highest FWP (1271.64 g) and the highest total tomato yield (FY) (40.69 t/ha). Meanwhile, tomato fruit yield was destructively affected by salt stress, showing minimum FWP (267.98 g) and total tomato yield (8.58 t/ha) in T_1_ treated plants (Fig. 2(A) and Fig. 2(B)). Other plants treated with manure-biochar compost showed similar improvements in FWP and total tomato yield and were represented in T_6_ (898.79 g and 28.76 t/ha, respectively), T_8_ (977.08 g, and 31.27 t/ha, respectively) and T_9_ (1172.91 g and 37.53 t/ha, respectively) treatment. The total FY under the MBC treatments increased by 235.40–374.53%, compared to absolute saline soil plants (Table 1). Manure-biochar compost-treated plants produced higher tomato yields than other organic amended plants under salt stress.

### Biomass yield of tomato plant

Manure-biochar compost and its organic matter had significant effects on above-ground biomass (FAGB), dry above-ground biomass (DAGB), fresh below-ground biomass (FBGB), dry below-ground biomass (DBGB), and total dry mass (TDM) (Table 3). Untreated saline soil showed significantly decreased tomato biomass yield. The organic amended plants showed higher fresh and dry biomass compared to abandoned saline soil (control treatment). The T_7_-treated tomato plant gave the maximum FAGB, FBGB, DAGB, DBGB, and TDM production (72.73 g, 18.84 g, 15.11 g, 7.61 g and 22.71 g, respectively). Like FAGB, FBGB, DAGB, DBGB, and TDM were produced in T_9_ (71.15 g, 18.56 g, 15.04 g, 7.30 g, and 22.34 g, respectively), T_8_ (69.32 g, 17.52 g, 14.05 g, 7.18 g, and 21.23 g, respectively) and T_6_ (68.20 g, 16.83 g, 13.03 g, 6.71 g, and 19.74 g, respectively) treatment. TDM was found significantly higher by 92.67–121.69%, under the MBC treatments over control plants (Table 1).

**Table 3:** Manure and biochar compost (MBC) effects on tomato plants cultivated in saline soil in terms of biomass yield.

Treatments	FAGB (g)	FBGB (g)	DAGB (g)	DBGB (g)	TDM (g)
T_1_	34.63 ± 1.80 c	9.68 ± 0.36 e	7.89 ± 0.95 e	2.36 ± 0.09 e	10.25 ± 1.04 g
T_2_	39.46 ± 3.76 c	12.47 ± 1.15 d	9.86 ± 0.57 de	4.32 ± 0.67 d	14.17 ± 0.46 f
T_3_	58.43 ± 1.86 b	15.75 ± 0.70 bc	12.92 ± 0.36 abc	6.39 ± 0.36 abc	19.31 ± 0.35 cd
T_4_	51.93 ± 1.40 b	13.99 ± 0.65 cd	10.87 ± 0.90 cd	5.59 ± 0.29 cd	16.46 ± 0.61 ef
T_5_	53.65 ± 5.52 b	14.35 ± 0.75 cd	11.83 ± 1.43 bcd	6.09 ± 0.22 bc	17.92 ± 1.21 de
T_6_	68.20 ± 2.48 a	16.83 ± 0.62 ab	13.03 ± 0.62 abc	6.71 ± 0.20 cde	19.74 ± 0.80 bcd
T_7_	72.73 ± 4.29 a	18,84 ± 1.17 a	15.11 ± 0.25 a	7.61 ± 0.94 a	22.71 ± 1.18 a
T_8_	69.32 ± 1.81 a	17.52 ± 0.78 ab	14.05 ± 0.57 ab	7.18 ± 0.17 ab	21.23 ± 0.43abc
T_9_	71.15 ± 2.78 a	18.56 ± 0.31 a	15.04 ± 1.06 a	7.30 ± 0.62 ab	22.34 ± 1.76ab
Significance level	< 0.001^***^	< 0.001^***^	< 0.001^***^	< 0.001^***^	< 0.001^***^

The value indicates the mean (± SD); P ≤ 0.05; n = 9; Different letters indicate significant differences among treatments by Tukey’s HSD test; FAGB = Fresh above-ground biomass, FBGB = Fresh below-ground biomass, DAGB = Dry above-ground biomass, DBGB = Dry below-ground biomass, TDM = Total dry mass; Treatments combinations are T 1  = SS; T 2  = SBTF; T 3  = B @ 3 t/ha + SBTF; T 4  = P @ 3 t/ha + SBTF; T 5  = C @ 3 t/ha + SBTF; T 6  = PBC (1:1) @ 3 t/ha + SBTF; T 7  = PBC (1:2) @ 3 t/ha + SBTF; T 8  = CBC (1:1) @ 3 t/ha + SBTF; T 9  = CBC (1:2) @ 3 t/ha + SBTF.

### Photosynthetic pigment content

The application of manure-biochar compost and its raw organic material increased the Chlorophyll (Ch) of salt-stressed tomato plants (Table 4). In comparison between both types of organic amendment applications (alone and compost), manure-biochar compost gave more positive results. SS stress significantly impaired the photosynthetic pigment of tomato plants and the lowest total Ch Ch *a*, and *b* contents were found in the T_1_ treatment (0.73, 0.51, and 0.22 mg/g, respectively). The application of different organic amendments considerably diminished the salt-stress impacts and improved pigmentation. In the T_7_ treated plant, the maximum total Ch and Ch *a* content (2.03 and 1.58 mg/g, respectively) were calculated. Likewise, salt-stressed plants in T_8_ (CBC (1:1) @ 3 t/ha + SBTF) showed the maximum Ch *b* (0.49 mg/g) content.

**Table 4:** Effects of manure and biochar compost (MBC) on the chlorophyll content of tomato leaf at 68 days after transplanting (DAT) grown in saline soil.  The value indicates the mean (± SD); *p* < 0. 05; n  = 9.

Treatments	Ch a (mg/g)	Ch b (mg/g)	Total Ch (mg/g)
T_1_	0.51 ± 0.11 f	0.22 ± 0.02 c	0.73 ± 0.13 e
T_2_	0.80 ± 0.12 e	0.28 ± 0.03 bc	1.08 ± 0.24 d
T_3_	1.30 ± 0.18 bcd	0.40 ± 0.10 ab	1.70 ± 0.23 bc
T_4_	1.11 ± 0.07 d	0.45 ± 0.08 a	1.57 ± 0.15 c
T_5_	1.18 ± 0.24 cd	0.48 ± 0.05 a	1.66 ± 0.20 c
T_6_	1.40 ± 0.04 abc	0.45 ± 0.07 a	1.85 ± 0.10 abc
T_7_	1.58 ± 0.05 a	0.45 ± 0.11 a	2.03 ± 0.09 a
T_8_	1.53 ± 0.13 ab	0.49 ± 0.16 a	2.02 ± 0.17 ab
T_9_	1.51 ± 0. 22 ab	0.46 ± 0.04 abc	1.97 ± 0.22 abc
Significance level	< 0.001^***^	˂ 0.021^*^	< 0.001^***^

Different letters indicate significant differences among treatments ( p  < 0.05) by Tukey’s HSD test. Treatments combinations are T 1  = SS; T 2  = SBTF; T 3  = B @ 3 t/ha + SBTF; T 4  = P @ 3 t/ha + SBTF; T 5  = C @ 3 t/ha + SBTF; T 6  = PBC (1:1) @ 3 t/ha + SBTF; T 7  = PBC (1:2) @ 3 t/ha + SBTF; T 8  = CBC (1:1) @ 3 t/ha + SBTF; T 9  = CBC (1:2) @ 3 t/ha + SBTF; Chlorophyll = Ch; chlorophyll a  = Ch a ; chlorophyll b  = Ch b.

### Response in osmoregulatory physiological traits

Salt stress considerably declined the RWC of tomato plant leaves, the lowest content in the T_1_ treatment (63.48%). Adding organic amendment plus SBTF fertilizer increased the RWC (%) of salt-stressed plant leaves, with the superiority of manure-biochar composted treatments (Table 5).

**Table 5:** Effects of manure and biochar compost (MBC) on RWC, water saturation deficit (WSD), and water retention capacity (WRC) of tomato plants at 65 DAT grown in saline soil. .

Treatments	RWC (%)	WSD (%)		WRC (%)
T_1_	63.48 ± 3.95 d	36.52 ± 3.95 a		10.90 ± 2.80 a
T_2_	70.74 ± 3.83 cd	29.26 ± 3 0.83 ab		9.54 ± 0.43 ab
T_3_	82.96 ± 3.18 ab	17.04 ± 3.18 cd		8.38 ± 0.39 bc
T_4_	79.54 ± 3.83 bc	20.46 ± 3.83 bc		8.64 ± 0.43 bc
T_5_	82.04 ± 1.97 ab	17.96 ± 1.97 cd		8.43 ± 0.55 bc
T_6_	85.72 ± 2.49 ab	14.28 ± 2.49 cd		8.03 ± 0.86 bc
T_7_	87.29 ± 3.25 ab	12.71 ± 3.25 d		8.28 ± 0.92 c
T_8_	86.69 ± 4.30ab	13.31 ± 4.30 cd		2.38 ± 0.15 c
T_9_	88.33 ± 1.95a	11.67 ± 1.95 cd		4.75 ± 0.44 c
Significance level	< 0.001^***^	< 0.001^***^		< 0.001^***^

The value indicates the mean (± SD); *p* < 0. 05; n  = 9. Different letters indicate significant differences among treatments ( p  < 0.05) by Tukey’s HSD test. Treatments combinations are T 1  = SS; T 2  = SBTF; T 3  = B @ 3 t/ha + SBTF; T 4  = P @ 3 t/ha + SBTF; T 5  = C @ 3 t/ha + SBTF; T 6  = PBC (1:1) @ 3 t/ha + SBTF; T 7  = PBC (1:2) @ 3 t/ha + SBTF; T 8  = CBC (1:1) @ 3 t/ha + SBTF; T 9  = CBC (1:2) @ 3 t/ha + SBTF.

The application of the T_9_ (CBC (1:2) @ 3 tha^−1^ + SBTF) treatment showed the highest retrieval of RWC (88.33%), followed by T_3_ (82.96%), T_6_ (85.72%), T_7_ (87.29%) and T_8_ (86.69%). In contrast, absolute SS treatment T_1_ showed significant increases in water saturation deficit (WSD) and water retention capacity (WRC) (36.52%, and 10.90%, respectively) (Table 5). In the organic amended T_9_ treatment, the lowest salinity-induced WSD (11.67%) and WRC were in T_8_ (2.38%) treatment (Table 5).

### **The concentration of H**_**2**_**O**_**2**_, **MDA**,** and electrolyte leakage (EL) in tomato plant leaf**

As shown in Table 6, H_2_O_2_, malondialdehyde (MDA) content, and electrolyte leakage (EL) in-tomato plant leaves were significantly affected by the SS and application of manure-biochar compost. The abandoned salinity treatment T_1_ treated plant leaves showed the significantly highest H_2_O_2_ and MDA contents (28.87 nmol/g and 15.49 nmol/g, respectively). However, the use of compost made of dung and charcoal significantly decreased their accumulation. The greatest reduction in H_2_O_2_ content was observed in the T_7_ amended plant, while the MDA content was reduced in T_9_ treated plants. Salt stress caused severe impairment of membrane stability by enhancing the electrolyte leakage (34.56%) in tomato plant leaves (Table 6). This reduction was significantly mitigated by the application of MBC amendment and SBTF-based fertilizer. Minimal electrolyte leakage (13.80%) was calculated for T_9_ treatment.

**Table 6:** Impact of manure and biochar compost (MBC) on H_2_O_2_, MDA, and electrolyte leakage (EL) of salt-stressed tomato plant leaves at 60 DAT.

Treatments	H_2_O_2_ (nmol/g)	MDA (nmol/g)	EL (%)
T_1_	28.87 ± 1.72 a	15.49 ± 1.67 a	34.56 ± 2.15 a
T_2_	24.19 ± 3.63 b	13.89 ± 0.85 ab	30.91 ± 1.43 ab
T_3_	14.98 ± 1.20 d	7.65 ± 1.20 cd	21.40 ± 0.66 cd
T_4_	19.38 ± 0.71 c	12.69 ± 1.70 ab	24.53 ± 2.09 bc
T_5_	20.02 ± 1.51 c	11.15 ± 1.86 bc	24.25 ± 1.20 bc
T_6_	12.48 ± 1.56 de	6.58 ± 0.91 d	17.51 ± 1.81 de
T_7_	10.77 ± 1.15 e	5.64 ± 0.15 d	15.18 ± 2.59 de
T_8_	13.76 ± 1.44 de	6.00 ± 0.57 d	17.26 ± 2.48 de
T_9_	12.18 ± 1.13 de	4.91 ± 0.62 d	13.80 ± 1.21 e
Significance level	< 0.001^***^	< 0.001^***^	< 0.001^***^

The value indicates the mean (± SD); *p* < 0. 05; n  = 9. Different letters indicate significant differences among treatments ( p  < 0.05) by Tukey’s HSD test. Treatments combinations are T 1  = SS; T 2  = SBTF; T 3  = B @ 3 t/ha + SBTF; T 4  = P @ 3 t/ha + SBTF; T 5  = C @ 3 t/ha + SBTF; T 6  = PBC (1:1) @ 3 t/ha + SBTF; T 7  = PBC (1:2) @ 3 t/ha + SBTF; T 8  = CBC (1:1) @ 3 t/ha + SBTF; T 9  = CBC (1:2) @ 3 t/ha + SBTF.

The statistically identical finding was recorded in treatment T_6_ (17.51%), T_7_ (15.18%), and T_8_ (17.26%). No significant improvement in electrolyte leakage was observed in plants treated with SBTF fertilizer alone.

### **Leaf proline**,** ascorbic acid**,** and soluble sugar content in tomato plant leaves**

Proline biosynthesis in tomato plants was activated by salt stress. The lowest proline accumulation (11.85 mg/g) was assessed from SS (T_1_) tomato plant leaves (Table 7). MBC plus SBTF-based fertilizer-amended tomato plant leaves had lower proline accretion under salt-stressed conditions. T_7_-treated plant leaves provided the maximum proline accretion (28.16 mg/g). Similar Statistics were obtained in T_6_, T_8_ and T_9_ amended plants (22.32, 21.95, and 26.86 mg/g, respectively). The application of various organic amendments significantly augmented the AA of the leaves of salinity-stressed tomato plants, among which MBC-treated plots performed better (Table 7). The application of the T_9_ [CBC (1:2) @ 3 t/ha + SBTF] treatment distinctly enhanced the ascorbic acid concentration (17.01 mg/g) of tomato plant leaves. SS (T_1_ treatment) plant leaves have the minimum amount of AA (8.48 mg/g). Similarly, the TSS content (21.63 mg/g) in tomato plant leaves decreased pointedly in abandoned salt-stress T_1_ treatment.

**Table 7:** Effects of manure and charcoal compost (MBC) on the levels of total soluble sugar (TSS), ascorbic acid (AA), and proline in the leaves growing in salty soil.

Treatments	Proline (mg/g)	Ascorbic acid (mg/g)	Total soluble sugar (mg/g)	
T_1_	11.85 ± 1.78 d	8.48 ± 1.53 d	21.63 ± 1.26 f	
T_2_	13.09 ± 1.93 d	9.42 ± 1.96 cd	22.74 ± 1.38 ef	
T_3_	18.36 ± 1.85 cd	13.69 ± 1.36 abc	27.64 ± 2.39 cde	
T_4_	17.02 ± 1.94 cd	12.60 ± 1.96 abcd	26.44 ± 1.37 def	
T_5_	16.84 ± 2.15 cd	11.81 ± 1.87 bcd	27.32 ± 2.09 cde	
T_6_	22.32 ± 3.01 abc	14.67 ± 2.44 ab	30.48 ± 2.40 cd	
T_7_	28.16 ± 1.57 a	15.71 ± 2.06 ab	36.94 ± 1.82 ab	
T_8_	21.95 ± 1.99 abc	14.39 ± 0.88 abc	32.02 ± 1.32 bc	
T_9_	26.86 ± 1.51 ab	17.01 ± 1.88 a	39.66 ± 2.70 a	
Significance level	< 0.001^***^	< 0.001^***^	< 0.001^***^	

The value indicates the mean (± SD); *p* < 0. 05; n  = 9. Different letters indicate significant differences among treatments ( p  < 0.05) by Tukey’s HSD test. Treatments combinations are T 1  = SS; T 2  = SBTF; T 3  = B @ 3 t/ha + SBTF; T 4  = P @ 3 t/ha + SBTF; T 5  = C @ 3 t/ha + SBTF; T 6  = PBC (1:1) @ 3 t/ha + SBTF; T 7  = PBC (1:2) @ 3 t/ha + SBTF; T 8  = CBC (1:1) @ 3 t/ha + SBTF; T 9  = CBC (1:2) @ 3 t/ha + SBTF.

MBC and its raw organic materials amended salt-stress tomato plant leaves showed a significant decline in total soluble sugar content. Plants grown in T_9_ (CBC (1:2) @ 3 t/ha + SBTF) amended saline soil produce the highest leaf TSS concentration (39.66 mg/g).

### Nutritional status of the tomato

Table 8 displays the results of the effects of MBC and its sole raw materials on the nutrients of tomato leaves under salt stress. Statistically noteworthy variances in the N, P, K, Na content, and K/Na ratio were found in organic amended tomato leaves. The N and P contents were highest in tomato leaves under T_7_ treatment (18.23 and 4.90 mg/g, respectively). While the T_9_-treated plant leaves in SS condition gave the maximum K content (14.80 mg/g). Similar leaves K concentrations were found in T_6_ (13.57 mg/g), T_7_ (14.74 mg/g) and T_8_ (13.30 mg/g) amended salt-stressed tomato plants. Untreated SS tomato plants in T_1_ treatment showed the lowest leaf nutrient contents of N, P, and K (9.29, 2.20, and 7.16 mg/g, respectively). Various organic amendments negatively influence the leaf Na accumulation. The T_9_ amended SS plant leaves exhibited the minimum Na concentration (6.03 mg/g), while the maximum Na content (12.47 mg/g) in T_1_ treatment.

**Table 8:** Manure and biochar compost (MBC) effects on tomato leaf nutritional content at 65 DAT in saline soil. .

Treatments	*N* (mg/g)	*P* (mg/g)	K (mg/g)	Na (mg/g)	Na/K ratio
T_1_	9.29 ± 1.38 b	2.20 ± 0.32 c	7.16 ± 1.58 e	17.47 ± 0.60 a	2.54 ± 0.67 a
T_2_	11.90 ± 1.57 ab	2.92 ± 0.54 bc	9.00 ± 0.59 de	15.00 ± 0.79 b	1.67 ± 0.79 b
T_3_	14.76 ± 2.53 ab	3.80 ± 0.37 abc	11.55 ± 0.44 bc	9.30 ± 1.02 cd	0.80 ± 0.07 c
T_4_	13.89 ± 1.64 ab	3.92 ± 0.66 abc	10.51 ± 0.59 cd	10.09 ± 0.31 c	0.96 ± 0.08 c
T_5_	14.23 ± 0.97 ab	4.04 ± 0.87 ab	10.30 ± 0.76 cd	10.28 ± 1.10 c	1.00 ± 0.10 bc
T_6_	17.34 ± 2.22 a	4.31 ± 0.36 ab	13.57 ± 0.68 ab	8.23 ± 0.23 cde	0.61 ± 0.03 c
T_7_	18.23 ± 1.03 a	4.90 ± 0.56 a	14.74 ± 0.40 a	6.35 ± 0.48 e	0.43 ± 0.03 c
T_8_	16.97 ± 1.76 a	4.14 ± 0.26 ab	13.30 ± 0.62 ab	7.07 ± 1.01 de	0.53 ± 0.06 c
T_9_	17.79 ± 2.61a	4.72 ± 0.39 ab	14.80 ± 0.18 a	6.03 ± 0.60 e	0.41 ± 0.04 c
Significance level	< 0.001^***^	< 0.001^***^	< 0.001^***^	< 0.001^***^	< 0.001^***^

The value indicates the mean (± SD); *p* < 0. 05; n  = 9. Different letters indicate significant differences among treatments ( p  < 0.05) by Tukey’s HSD test. Treatments combinations are T 1  = SS; T 2  = SBTF; T 3  = B @ 3 t/ha + SBTF; T 4  = P @ 3 t/ha + SBTF; T 5  = C @ 3 t/ha + SBTF; T 6  = PBC (1:1) @ 3 t/ha + SBTF; T 7  = PBC (1:2) @ 3 t/ha + SBTF; T 8  = CBC (1:1) @ 3 t/ha + SBTF; T 9  = CBC (1:2) @ 3 t/ha + SBTF.

The K/Na ratio of manure-biochar compost and its raw material and/or SBTF-fertilized plants increased compared to abandoned saline soils (Table 8).

## Discussion

One of the biggest risks to crop growth is soil salinization, which lowers crop yields significantly all over the world^60^. The findings demonstrated that tomato plant development and biomass productivity were significantly reduced by soil salinization (Fig. 1; Table 3). This might be because low turgor pressure brought on by salinity prevents cells from expanding, which lowers shoot development and growth. Elevated salt stress has the potential to trigger the synthesis of inhibitors such as abscisic acid and impede the development of growth promoters for plants^61^. Furthermore, salinity inhibits root system growth, altering its morphology and physiology. These modifications result in altered water and ion absorption, ultimately reducing plant growth^62^. The decrease in compound leaves and leaf area may be due to the salt-induced osmotic effect, which reduces the availability of water and nutrients to the roots and eventually disturbs the plant tissues. This would lead to a decrease in meristematic tissue activity and cell expansion^63^. Merely applying chemical fertilizer cannot compensate for the decrease in biomass production brought on by salt. The application of MBC and its organic components reduced the impacts of salt stress and led to a noteworthy increase in the vegetative attributes of tomato plants under SS stress, including PH, NBP, NLP, LA, and biomass, demonstrating its processes of mitigating salt stress (Figs. 1 and 2; Table 3). The growth and biomass output of tomato plants under salt stress were enhanced by the application of organic amendments, which also increased the soil’s organic matter and increased hydraulic conductivity, nutrient availability, soil bulk density, and soil water hold capacity^64^. Improved ionic balance and stressed plants’ physiological efficiency are guaranteed by increased leaf proliferation in tomato plants grown in salinized soil amendments^65^. Noteworthy differences among traits were demonstrated for ANOVA of our study which were corroborative to the results of agronomic traits of lentil^66^, field pea^67^, mungbean^68,69^, *Zea* mays^70,71^, *Oryza sativa*^72–77^, *Amaranthus* spp^78,79^.

Under salt stress, tomato plant leaves’ photosynthetic pigments were less effective (Table 4). However, because SS denaturates the enzymes that synthesize chlorophyll content, it produces oxidative stress in chloroplasts and lowers chlorophyll content^80^. Because of the salt-induced osmotic imbalance in this investigation, SS dramatically decreased leaf RWC (Table 5), which in turn reduced the tomato plants WSD and WUC. However, the application of MBC greatly boosted the leaf RWC. This could be because organic amendments have a stronger ameliorating effect and create ideal soil conditions for higher water absorption under SS. Salt stress positively enhanced the oxidative stress markers MDA and H_2_O_2_ in tomato plants (Table 6). High accumulation of H_2_O_2_ induces oxidative stress, which disrupts plant physiological processes, leading to growth and yield losses. SS directly affects higher membrane damage, called MDA accumulation, which plays a crucial role in increasing the EL of tomato plant leaves (Table 6). SS increases ROS production, thereby damaging cell membranes and causing a significant increase in EL^81^. The substantial reduction in EL in MBC-treated tomato plants indicates a better-alleviating effect of compost treatment, which was associated with lower MDA and H_2_O_2_ (Table 6)^2^. Under SS, various organic amendments reduced ROS production, ultimately alleviating MDA accusation by reducing oxidative stress in tomato plants^1^. Tomato plants regulate and increase osmolyte biosynthesis and maintain Na^+^ refflux to counteract the toxic effects of salt stress^64,82^. In the current study, the concentration of proline, soluble sugar, and ascorbic acid in tomato leaves of salt-stressed tomato plants was lower, indicating lower adaptability (Table 7). A key component of plants’ defense mechanisms against salt stress is osmoregulation. Under salt stress, plants create osmotically active solutes (like proline) to promote protein biosynthesis or metabolism^26^ and balance water potential^83^ to protect themselves from abiotic stress. To control osmotic pressure, a significant amount of osmolytes, such as soluble carbohydrates and proline, build up in the cytoplasm and other organelles^59^. The application of MBC resulted in higher proline biosynthesis in the leaves of tomato plants exposed to SS (Table 7), suggesting that balanced osmosis protects their photosynthetic machinery. In addition, amended tomato plants can accumulate larger amounts of proline to scavenge ROS, reduce oxidative damage, and protect cell membranes from the negative effects of salt stress.

Higher proline accumulation in MBC-treated soil may be due to salt-induced increases in N content and metabolism^84^. Under saline conditions, MBC and its raw materials increased the content of alternative important osmolytes, namely TSS, in the tomato plant leaves (Table 7). A substantial increase in sugar accumulation in amended plants indicates effective protection of tomato plants exposed to salt stress. Total soluble sugar plays key roles in many physiological and biochemical processes, including chlorophyll content, scavenging of ROS, and induction of damaging salt-stress conditions in adaptive pathways^85^, Studies have shown that proline and ascorbic acid are potent antioxidants that can scavenge various types of ROS and protect cells from oxidative damage^86^. Salt-stressed tomato plants treated with MBC showed higher ascorbic acid (AsA) accumulation (Table 7). A higher concentration of AsA indicates its better detoxification capacity against salt-induced ROS production.

Salt stress exerts a notable inhibitory effect on various physiological processes, including seed germination, lateral root growth, and biomass generation, leading to significant yield loss. A significant decline in the morphological, physiological, and biochemical indices of watermelon seedlings exposed to salt stress^87^. Salt stress persuaded higher concentration of Na content in tomato plant leaves but lessened other nutrients like N, P, and K contents (Table 8). The application of organic amendments creates provision for larger N, P, and K uptake, and diminishes the salt-induced harmful amount of Na uptake in tomato leaves (Table 8). Osmotic stress is a common secondary stress of NaCl salinity in many plants that can negatively influence many aspects of plant metabolisms^88,89^. The accumulation of large amounts of Na in plant tissues exposed to saline conditions can have damaging effects on the metabolism of cytoplasm and organelles^64^. Excess Na causes an imbalance in cellular Na and K homeostasis, often resulting in a low Na/K ratio^90^. Maintaining a low Na/K ratio in leaves is an important feature of plant salt tolerance^91^. Under salinity stress, the Na/K ratio increased sharply due to excess Na uptake in the leaves. These results indicate that MBC and other organic amendments can promote nutrient balance and reduce ion toxicity thereby accelerating the vegetative development of plants. One practical method of improving salt tolerance is by high intake of mineral nutrients^92^. Biochar addition reduces plant sodium uptake in salt-stressed soils due to its high uptake capacity through transient binding of Na^+^ and release of mineral nutrients (K^+^, Ca^2+^, Mg^2+^ particles) into solution^93,94^. Therefore, it can be concluded that biochar prevents sodium uptake by plants by releasing nutrients into the soil solution. Biochar might have the potential to release large amounts of Ca and Mg and displace Na^+^ at soil exchange sites, thereby reducing the availability of Na^+^ to plants^95^. Since BC has a larger surface area, CEC, and porosity, the addition of BC hinders the absorption and accumulation of Na^+96^. BC application increases Ca^2+^ content, thereby improving SS tolerance by altering cell signaling pathways^97^. The application of biochar can reduce the Na/K ratio and Ca^2+^ through the Ca^2+^ dependent SOS pathway^98^. Sodium elimination in roots occurs primarily through the plasma membrane Na^+^/H^+^ antiporter, a well-known gene (SOS1)^96^. Overexpression of vacuolar Na^+^/H^+^ antiporters increase salt tolerance in plants^99^. Adding biochar to soil improves electrochemical properties, such as root zeta potential, thereby increasing nutrient uptake by plants^100^. The suppressing effect of vermicompost as well as biochar might be attributed to the release of toxic compounds such as ammonium and improved soil nutrient and plant growth, leading to plants more tolerant to nematode damage^101^. In addition, the use of manure- biochar compost has a significant positive effect on improving the growth of microorganisms and the release and uptake of nutrients in saline soil, providing a practical option for alleviating salt stress in tomato plant^102^. The presence of plant nutrients and ash in the biochar and its large surface area, porous nature, and the ability to act as a medium for microorganisms have been identified as the main reasons for the improvement in soil properties and increase in the absorption of nutrients by plants in soils treated with biochar^103^. The positive effects of biochar on the interactions between soil-plant-water caused better photosynthetic performance and improved nitrogen and water use efficiency also biochar has the potential to improve the properties of soil, microbial abundance, biological nitrogen fixation, and plant growth. Therefore, it is recommended to use biochar as a soil amendment for long-term carbon sink restoration^104^. These biochars acted as a nutrient source as well as a suitable cation exchanger due to their large surface area resulting in the availability of nutrients and their higher uptake by plant roots^105^.Under SS stress, plants display disturbances in several physiological processes, leading to a decrease in tomato production (i.e., SFW and TFWP) (Fig. 2) and yield attributes (i.e., NFCP, NFC) (Table 2). Tomato output is significantly reduced by SS because it damages photosynthetic pigments, results in ionic imbalance and ROS generation, and decreases nutrient uptake, RWC, and membrane integrity ^106,107^. By altering chlorophyll pigments, salinity prevents the buildup of photoassimilates, which lowers tomato output characteristics and, in turn, lowers fruit weight^108^. But when MBC and its organic materials were applied, tomato plants under salt stress produced much more, suggesting that salt stress can be lessened (Table 2; Fig. 2). The previous research demonstrates that the use of organic amendment enhanced the productivity and yield characteristics of tomato plants^109^ by improving the physiological mechanism of osmoregulation, photosynthetic efficiency, nutrient uptake, and antioxidant activities, K uptake, and Na/K reduction. Moreover, organic matter enhances the qualities of the soil and provides plants with greater capacity for water absorption, photosynthetic pigments, and biochemical activity. This increases photosynthesis, which is positively correlated with increased fruit yield characteristics and fruit weight^110^. In addition to organic amendment, literature have shown that K amendment increased yield, quality, and drought tolerance in soybean^111^, and mung bean growth, yield, nutrient content, and drought tolerance^112^. Nano iron oxide accelerates growth, yield, and quality of soybean^113^.

In summary, biochar is the best organic amendment to act as a source of soil nutrients for improved tomato growth, crop yield, and environmental benefits. SS lowers photosynthetic pigments, nutrient uptake, and leaf water content while increasing H_2_O_2_, MDA, and Na accumulation. These effects ultimately lower tomato output and plant growth. According to our findings, under salt stress, MBC and its organic matter can enhance tomato output and plant growth. The salt-treated saline soil (PBC (1:2) @ 3 tha^−1^ + SBTF) had the highest biomass and tomato fruit production. Based on how much salt stress reduces tomato yield, T7 > T9 > T8 > T6 is the order in which MBC treatments are applied. To decrease leaf oxidative damage, MBC was applied. This resulted in an increase in chlorophyll content, leaves’ RWC, nutrient absorption, Na/K ratio, antioxidant activity, and osmoregulatory characteristics. MBC also lessened the generation of ROS and Na buildup. Consequently, MBC exhibits its tolerance mechanism to salt stress and has a good impact on tomato plants’ growth characteristics, photosynthetic mechanism, and physiological and osmotic adaptation features. Therefore, the combined use of biochar and compost can be beneficial in reducing the detrimental effects of salinity on horticultural crops and increasing tomato productivity in salt-affected soils. Based on our findings, additional research is suggested to explore potential mechanisms involved in the improvement of physiology, growth, and productivity under salt stress.

## Methods

### Planting materials and systems for growth

The investigation was led in a salinity-affected farmer’s field in Batiaghata Union, Khulna (22° 41΄ 36.1˝ N and 89° 31’56. 0˝ E) from December 2018 to March 2019. The site is located under the AEZ of the Ganges tidal floodplain (AEZ 13). The high-yielding indeterminate tomato (*Lycopersicon esculentum* L.) cultivar BARI Tomato-9 (Lalima) was used. Tomato seeds were surface sterilized with 70% ethanol solution and 5% sodium hypochlorite solution (NaOCl) for 15 min, and then washed with distilled water. For two days, tomato seeds were grown on damp filter paper in the dark. Then sown in sand-filled trays and grown under the transparent plastic shed for 10 d. At the second-leaf stage, the seedlings were uprooted and placed in plastic bags filled with soil, grown for 20 d, and then transplanted to the experimental field. With soil salinity i.e., ECe (6.62 dS/m), pH (7.52), organic carbon (OC) (0.68%), total nitrogen (TN) (0.09%), available phosphorus (P) (18.40 mg kg^−1^), and exchangeable Ca, Mg, Na, and K (7.44, 3.72, 18.67, and 0.27 cmol kg^−1^), respectively, the soil belonged to the silty clay loam textural class (sand 18.10%, silt 45.30%, and clay 36.60%) (Table 9). The average daily temperature, minimum temperature, and relative humidity for the tomato cultivation season were T max = 34.3 °C and T min = 26.2 °C; RH max = 95.1% and RH min = 67.3%, respectively. In accordance with our institution’s, as well as national and international norms and laws, experimental research, laboratory, and field investigations of the effects of salinity stress on organic amendments in tomatoes, including the collecting of tomato seeds, were conducted.

**Table 9:** Physico-chemical features of the research field soil.

Composition (% (w/w))			pH	ECe	OC	TN	*P*	K	Ca	Mg	Na
Sand	Silt	Clay		dS m^-1^	(%)	(%)	(mg kg^-1^)	(cmol kg^-1^)	(cmol kg^-1^)	(cmol kg^-1^)	(cmol kg^-1^)
18.10	45.3	36.60	7.52	6.62	0.68	0.09	18.40	0.27	7.46	3.72	18.67

ECe = Electrical conductivity; OC = Organic carbon; TN = Total nitrogen; P = Phosphorus; K = Potassium; Ca = Calcium; Mg = Magnesium; Na = Sodium.

### Manure-biochar compost preparation

Biochar used in field experiments was obtained by pyrolysis of rice straw in a two-chamber pyrolysis furnace at 400–500 °C. Locally collected cow dung and poultry manure were kept in ambient conditions for a week to lessen extra moisture. Table 10 displays the chemical characteristics of the additional organic compounds.

**Table 10:** Chemical properties of different applications of organic substances.

OM	pH	OC (%)	Total *N* (%)	*P* (%)	K (%)	Ca (%)	Mg (%)	Zn (mg kg^−1^)
Cow dung	7.28	22.94	1.13	0.35	0.80	0.97	0.54	142.10
Poultry manure	8.02	34.20	1.45	0.73	0.97	1.51	0.51	178.10
Biochar (Rice straw)	7.65	44.03	0.86	0.34	2.84	1.68	0.49	240.10
PBC (1:1)	7.78	39.12	1.81	0.57	0.98	1.55	0.50	153.50
PBC (1:2)	7.67	39.72	1.94	0.46	0.95	1.67	0.50	194.30
CBC (1:1)	7.37	34.49	1.53	0.41	0.87	1.49	0.56	198.10
CBC (1:2)	7.51	38.66	1.76	0.38	0.86	1.63	0.49	212.43

*OC* = Organic carbon; *N* = Nitrogen; *P* = Phosphorus; *K* = Potassium; *Ca* = Calcium; *Mg* = Magnesium; *Zn* = Zinc; *PBC* = poultry manure-biochar compost; *CBC* = cow dung-biochar compost.

For compost preparation, manuring substances (M) (cow dung (C) and poultry manure (P)) and biochar (B) were mixed at the ratio of 1:1 (M: BC, v/v) and 1:2 (M: BC, v/v). After 6 weeks cow dung-biochar compost (CBC) and poultry manure-biochar compost (PBC) were ready for field application. The produced MBC is thoroughly mixed before use as a soil amendment.

### Experimentation

Abandoned salinized land was treated with different manure-biochar compost and their raw organic substances. Three different organic amendment treatments and their compost mixtures [CBC (1:1; and 1:2 v/v ratio)] and [PBC (1:1; and 1:2 v/v ratio)] plus SBTF dose were evaluated. For comparison, we used reclaimed salinized soil and sole SBTF-based chemical fertilizer-treated plots. The treatments are: T_1_ = absolute SS; T_2_ = SBTF dose; T_3_ = biochar (B) @ 3 t/ha + SBTF; T_4_ = poultry manure (P) @ 3 t/ha + SBTF; T_5_ = cow dung (C) @ 3 tha^−1^ + SBTF; T_6_ = PBC (1:1) @ 3 t/ha + SBTF; T_7_ = PBC (1:2) @ 3 t/ha + SBTF; T_8_ = CBC (1:1) @ 3 t/ha + SBTF; T_9_ = CBC (1:2) @ 3 t/ha + SBTF. Three replications of a randomized complete block design (RCBD) were employed to order the treatments in the field study. A drainage system of 2.5 × 2.0 m and 0.5 m was built into every plot. On December 5, 2018, four-week-old tomato seedlings were transplanted, with 60 cm × 50 cm spacing between each seedling and two seedlings per hill. The same seedlings were used to repair the gaps one week later. Fertilizers based on SBTF were used to fertilize the crop. computed using Fertilizer Recommendation Guide^114^ to yield 321 kg of urea, 85 kg of triple superphosphate (TSP), 22 kg of muriate of potash (MP), and 20 kg of gypsum per ha^−1^. Final land preparation included basal application of one-third of urea, TSP, MP, gypsum, and full volumes of organic amendments. Two top dressing applications of the leftover computed urea were made at 15 and 35 days after transplantation (DAT). We maintained the field capacity (FC) of the soil (moisture content of soil 30.7%) for each pot. Irrigation was provided at 12 h intervals. The moisture loss every day was fulfilled by adding tap water using a moisture meter until the soil moisture level was raised to 30.7%. As we maintained FC (water only in the micropores of the soil), so very minimal water was required every day to maintain FC and there was no leaching or percolation of the potting soil. As a result, the salinity of the soil remains constant throughout the cropping season. The conventional cultivation method used in Bangladesh was followed when applying tomato production to agriculture.

### Tomato growth and yield characteristics are measured

During the active flowering period at 65 DAT, several growth parameters [PH (cm), NBP, NLP, and LA] were assessed. Yield characteristics of tomato fruit (NEFC and NFC) were counted in three selected plants and their average value was calculated. Tomato fruit yield was calculated by counting NFP and SFW and weighting all the picked fruits collected at the ripening stage from each plant. Fruits were harvested four (4) times during the harvesting time and that began on 13 March 2019. In the senescence stage, the whole plant, fresh weight (FW), and dry weight (DW) were calculated to obtain biomass yield. To determine the total dry mass (TDM), plants were divided into shoots and roots and air-dried first at room temperature and finally oven-dried at 65 °C for 72 h.

### Measurement of pigment content

Using the method outlined by Lichtenthaler et al.^115^, the total chlorophyll content (mg/g FW) was determined from the completely prolonged top most leaf at the flowering stage (67 DAT). To extract leaf chlorophyll, 20 mg of completely inflated leaf samples were taken, put in tubes covered in aluminum foil with 20 mL of 80% acetone, and left in the dark for the entire night at 4 °C. Following centrifugation, a spectrophotometer was used to measure the absorbance at 645 nm and 663 nm wavelengths.

### Measurement of electrolyte leakage and water status

The Sullivan^116^ method was used to measure the electrolyte loss in the leaves at 67 days after harvest. Leaf samples weighing about 0.5 g were collected and placed in two distinct test tube sets, each holding 10 mL of deionized water. The first group was incubated for ten minutes at room temperature, while the second set spent thirty minutes in a water bath at fifty-five degrees. An EC meter was used to determine the electrical conductivity (EC) of the supernatants from both sets. ECa and ECb are the names of the first and second sets of ECs, respectively. The following formula was used to determine the electrolyte leakage:

Electrolyte leakage (%) = {(EC_b_ - EC_a_)/EC_e_} × 100.

The technique of Hayat et al.^117^ was used to calculate the leaf relative water content (RWC). Samples of fully-grown leaves (at 67 DAT) were collected, and the fresh weight (FW) was measured. To determine the turgid weight (TW), the same leaves were immersed in distilled water for four hours. For 72 h, the leaf samples were oven-dried at 70 °C to obtain a consistent dry weight (DW). The formula used to compute the RWC of leaves was: - RWC (%) = {(FW – DW)/ (TW – DW)} × 100.

As per Sangakkara et al.^118^, the following formulas were used to compute the water saturation deficit (WSD), water retention capacity (WRC), and water uptake capacity (WUC). Water saturation deficit (WSD) = {(TW – FW)/(TW – DW)} × 100.

Water retention capacity (WRC) = (TW/DW).

Where, FW = Fresh weight (mg), DW = Dry weight (mg) and TW = Turgid weight (mg).

### Determination of malondialdehyde (MDA) and H_2_O_2_concentrations

The H_2_O_2_ content (nmol/g FW) in plant samples was determined by the method of Su and Silva^119^. Fresh plant leaf sample (0.5 g) was extracted with 5 mL of 5% (w/v) trichloric acid (TCA) and centrifuged at 6000 × *g* for 15 min. The reaction mixture consisted of 1 mL 1.0 M potassium iodide (KI) and 100 µL of 10 mM potassium phosphate buffer (pH 7.0). Then the content of H_2_O_2_ was determined by using a spectrophotometer at 390 nm wavelength from a standard curve.

The assay for thiobarbituric acid (TBA) was utilized to ascertain the MDA concentration (nmol/g FW)^119^. To extract MDA, a 0.5 g leaf sample was taken, homogenized in 5.0 mL of 5% (w/v) trichloroacetic acid (TCA), and centrifuged for 10 min at 12,000 × *g*. After that, 1 mL of supernatant was added to the reaction mixer, which held 4 mL of 20% TCA with 0.5% TBA, and it was maintained in a water bath at 95 °C for 30 min. To find the MDA content, absorbance measurements were made at 532, 600, and 450 nm following cooling in an ice bath. The following formula was used to determine the tomato leaf’s MDA content: 0.56 × A450–6.45 × (A532 – A600).

### Determination of proline, ascorbic acid, and total soluble sugar content

The method outlined by Bates et al.^120^. was utilized to measure the proline content (mg/g FW) of leaves. Leaf samples (0.5 g) were pulverized in 10 mL of 3% (v/v) sulfosalicylic acid and centrifuged at 10,000 × g for 10 min to extract proline from leaves (67 DAT). Two milliliters of the supernatant (2 mL) were added to a test tube along with two milliliters each of glacial acetic acid and acid-ninhydrin solution. After 30 min of incubation in a water bath at 100 °C, the mixer was cooled in an ice bath. To separate the toluene and aqueous phases, 4 mL of toluene was added to the reaction mixture after it had cooled. It was then let to stand in the dark at room temperature for 20 min. After that, the toluene phase was meticulously collected, and a spectrophotometer was used to measure the absorbance at 520 nm. Analytical proline was used to generate a standard curve that showed the concentration of free proline.

The ascorbic acid (AA) content (mg/g FW) was calculated utilizing the method that Jagota and Dani^121^ outlined. A 0.5 g leaf sample was weighed, homogenized in 2 mL of 5% trichloroacetic acid (TCA), and centrifuged at 10,000 × g for 15 min at 4 °C to produce the AA extraction. Next, 8 mL of 10% TCA was added to 2 mL of supernatant that had been collected in a test tube. After giving the mixer a good shake, it was put in an ice bath for five minutes, and it was centrifuged once more for five minutes at 3000 × g. 5 mL of the extract was combined with 2 mL of distilled water diluted with 2 mL of Folin-Ciocalteu reagent, and the mixture was left for 10 min to develop the blue color. At 760 nm, the AA content was determined by spectrophotometry.

Leaf total soluble-sugar content (mg/g FW) was determined using a colorimetric method^122^. Fresh leaf sample was collected and homogenized, then extracted with water at room temperature. Phenol and sulfuric acid are then added to prepare the extract. Total soluble sugar content was measured with a spectrophotometer at 485 nm.

### Determination of nutrient contents in leaf

The N, P, K, Ca, and Na contents (mg/g DW) of each plant’s growing third and fourth leaves at the flowering stage (67 DAT) were measured. A 20-mesh sieve was used to grind and homogenize the oven-dried leaf samples in preparation for analysis. The micro Kjeldahl method was used to calculate the sample of leaves’ total nitrogen content. A 0.2 g plant sample was digested with concentrated H_2_SO_4_ at 360 °C for two hours while a catalyst mixture (K_2_SO_4_: CuSO_4_.5H_2_O: Se = 100:10:1) was present to determine the amount of N present. Then the digests were distilled off in an auto distillation unit using 40% NaOH solution and collected in 4% H_3_BO_4_; finally titrated with auto burette against 0.02 N (N/50) H_2_SO_4_ and calculated^123^. For the determination of nutrient element concentrations except for nitrogen, 0.5 g ground leaf samples were digested with the di-acid mixture (HNO_3_: HClO_4_ = 5:1) heated on a sand bath at 130 °C until colorless fume evolved^124^. Leaf phosphorus content was determined by the ammonium molybdovanadate method using a spectrophotometer^125^. Leaf potassium ion (K^+^), calcium ion (Ca^2+^), and sodium ion (Na^+^) contents were assessed with the help of an atomic absorption spectrophotometer (Model No 170 − 30, HITACHI, Japan)^126^.

### Statistical analysis

Trait-wise average dta were analyzed statistically^127,128^ and biometrically^129,130^. The analysis of variance (ANOVA) test was run on the experimental data using the SPSS statistical tool (SPSS 16.0, SPSS Inc., USA). Tukey’s HSD Test was utilized to determine the significance of variation across treatments.

## Electronic supplementary material

Below is the link to the electronic supplementary material.

Supplementary Material 1

## Data Availability

Data is provided within the manuscript or supplementary information files.
